# Advances in research on thermogenic substances

**DOI:** 10.1186/s40101-026-00428-8

**Published:** 2026-04-17

**Authors:** Mengdi Tao, Yutong Gao, Xuxia Chai, Jiahui Peng, Changjiang Guo, Lixia Yu, Zhanxin Yao

**Affiliations:** 1 Military Medical Sciences Academy, Academy of Military Sciences, Tianjin, 300050 China; 2 Shanghai Ocean University, Shanghai, 201306 China; 3 Tianjin University of Traditional Chinese Medicine, Tianjin, 301617 China; 4 Tianjin Medical University, Tianjin, 300070 China

**Keywords:** Thermogenesis, Nutrients, Traditional Chinese medicine, Brown adipose tissue, Uncoupling protein 1

## Abstract

In mammals, a constant body temperature is maintained for survival in cold environments, primarily through the activation of thermogenic mechanisms. In this review, we discussed the advances in research on cold-adaptive thermogenic substances and their regulation. First, we outlined the primary thermogenic responses to cold exposure, including shivering thermogenesis (ST) and non-shivering thermogenesis (NST), and emerging thermogenic pathways. Next, we discussed the mechanism of action of various thermogenic substances, including nutrients, traditional Chinese medicine (TCM), TCM extracts, and dietary functional factors. This review provides a theoretical foundation for developing cold-resistance preparations with minimal side effects.

## Introduction

Exposure to low temperatures threatens human health. Prolonged or extreme low temperatures disrupt the balance between heat production and dissipation in the body, consequently inducing hypothermia, cardiovascular dysfunction, metabolic dysregulation, and other complications. To counteract the adverse effects of cold stress, mammals have evolved an efficient cold-defense mechanism centered on non-shivering thermogenesis in brown adipose tissue (BAT). Exogenous thermogenic agents, such as naturally derived nutrients, bioactive compounds from traditional Chinese medicines, and dietary functional factors, promote cold adaptation due to their low toxicity, multi-pathway synergies, and innate compatibility with the metabolic network in organisms.

## Biological basis of thermogenic mechanisms and cold adaptation

### Shivering thermogenesis

Shivering thermogenesis (ST) is a mechanism that is rapidly activated in endothermic animals to cope with low-temperature stress. During ST, chemical energy is converted into thermal energy through autonomous high-frequency contractions (shivering) of skeletal muscles [1]. The mechanism of action involves the activation of skeletal muscle motor units by the hypothalamic thermoregulatory center via sympathetic nerves, triggering high-frequency microtremors in muscle fibers. ATP is quickly generated by muscle cells through glycolysis and mitochondrial oxidative phosphorylation, with approximately 70% of the energy released as heat. However, ST has certain physiological limitations—while heat is generated rapidly, sustained shivering leads to muscle fatigue, and the low energy efficiency of ST makes the process unsustainable for long-term maintenance of body temperature.

### Non-shivering thermogenesis

#### Classic pathway: UCP1-mediated mitochondrial uncoupling in thermogenesis

Non-shivering thermogenesis (NST) is the core mechanism underlying long-term cold adaptation. NST primarily occurs in BAT via uncoupling protein 1 (UCP1), which uncouples oxidative phosphorylation to directly convert proton gradient energy into thermal energy. With the same energy expenditure, the heat produced by NST is 3 to 5 times that of ST. NST can sustainably enhance the thermogenic capacity by adaptively increasing BAT mass (adipose browning) [2–4].

The transmembrane protein UCP1 is a member of the mitochondrial anion carrier (SLC25) family and is located on the inner mitochondrial membrane of brown and beige adipocytes. It consists of six α-helical transmembrane domains that form a proton channel. UCP1 serves as the core molecule of NST, primarily relying on the proton gradient across the inner mitochondrial membrane to allow protons to bypass ATP synthase and flow back directly into the mitochondrial matrix. This process uncouples oxidative phosphorylation, leading to the dissipation of energy as heat [2, 5–8]. The thermogenic capacity of BAT is attributed to its abundance of mitochondria, high expression of UCP1, and multilocular lipid droplets, which collectively impart defense against low temperature [6, 9]. PGC-1α is a master regulator of mitochondrial biogenesis. Exposure to cold or stimulation of the β3-adrenergic receptor induces the expression of PPARγ coactivator-1α (PGC-1α), which in turn activates multiple transcription factors, resulting in an increase in the expression of Ucp1 and mitochondrial genes [10, 11]. Cold stress also activates the AMPK signaling pathway, promoting mitochondrial biogenesis in BAT, improving mitochondrial function, increasing thermogenic capacity, and ultimately maintaining body temperature homeostasis [12–14]. Additionally, BAT-like cells in white adipose depots, known as beige adipocytes, can acquire thermogenic capabilities similar to BAT when stimulated by low temperature or exercise. This phenomenon is known as “browning.” The process is regulated by the transcription factors PRDM16, PPARγ, and PGC-1α, which together promote the expression of UCP1 and other thermogenic proteins [15–20].

**Fig. 1:**
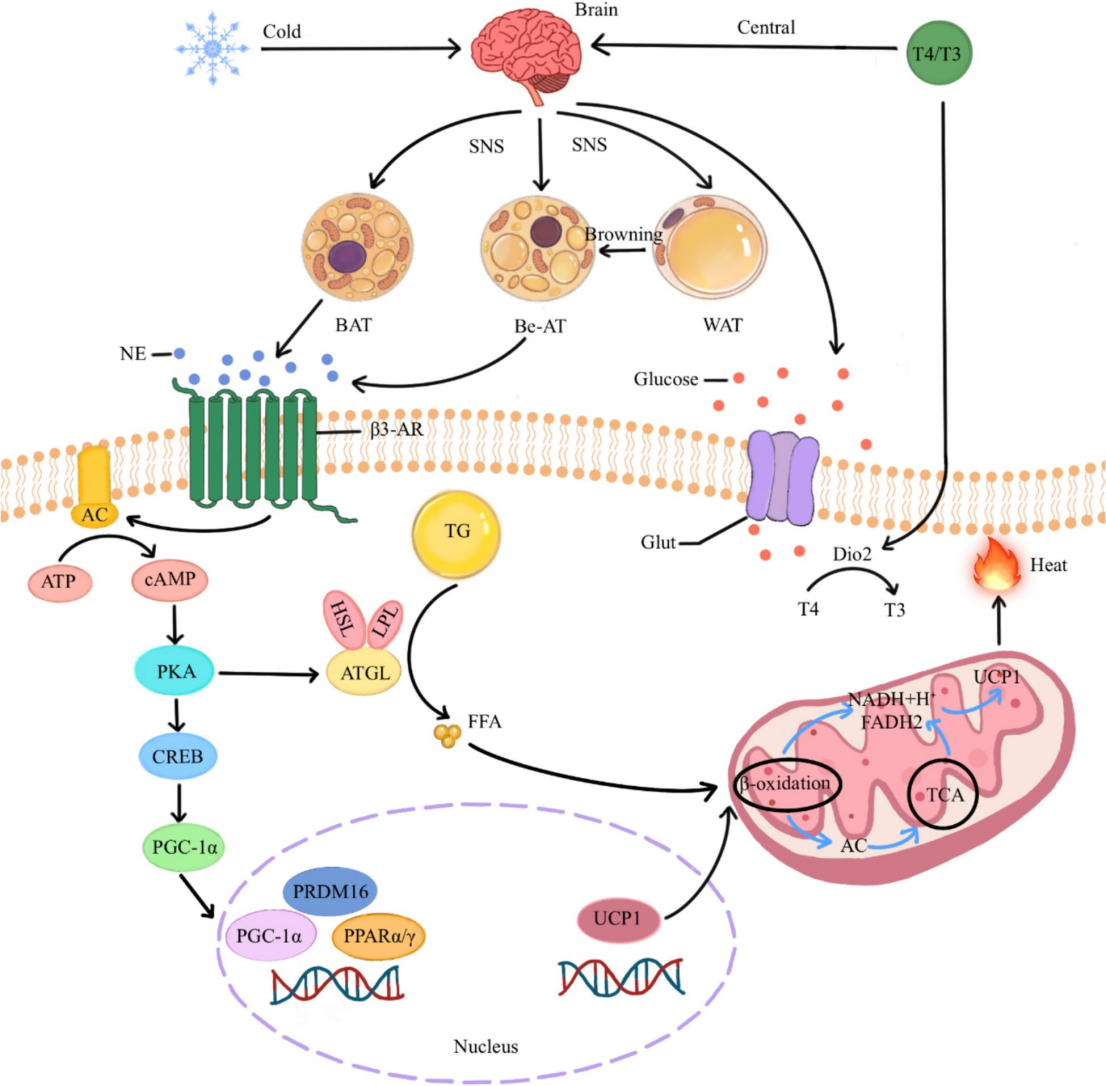
The mechanism underlying UCP1-mediated mitochondrial uncoupling thermogenesis

Brown and beige adipocytes exhibit their thermogenic capacity through the uncoupled oxidation of glucose and lipids, which results in heat production. The process is as follows (Fig. 1). In the resting state, the proton conductance activity of UCP1 on the inner mitochondrial membrane is inhibited by cytosolic purine nucleotides, particularly ATP [21]. After BAT is activated by ambient temperature changes or alterations in body energy stores via central neural pathways, it responds to the release of sympathetic norepinephrine. This catecholamine stimulates the β-adrenergic receptor/Gs protein/cAMP cascade in brown adipocytes, subsequently activating the PKA-CREB signaling pathway to induce the expression of the *UCP1* gene and lipolysis [22]. Triglycerides stored in the lipid droplets of mature brown adipocytes undergo hydrolysis mediated by lipolytic enzymes (HSL, ATGL, and LPL), liberating free fatty acids (FFAs) that serve as activators of UCP1 and substrates for mitochondrial β-oxidation, ultimately dissipating chemical energy as heat through uncoupled respiration [23, 24]. FFAs also undergo β-oxidation to form acetyl coenzyme A, which enters the tricarboxylic acid cycle and is oxidized to form nicotinamide adenine dinucleotide (NADH) and flavin adenine dinucleotide (FADH_2_). The electron transport system uses NADH and FADH_2_ to generate a proton gradient.

In the sympathetic nervous system (SNS), a major division of the autonomic nervous system (ANS), catecholaminergic neurons secrete norepinephrine (NE) to regulate different types of peripheral tissues and organs [25]. BAT receives dense sympathetic innervation, whose activation serves as the primary physiological stimulus for the thermogenic activity of BAT. Sympathetically released NE stimulates the hydrolysis of triglycerides (TGs) in brown adipocytes, liberating long-chain fatty acids that activate UCP1-mediated thermogenesis [26]. Exposure to chronic cold enhances the sympathetic tone, promoting the expansion of BAT and white adipose tissue (WAT) browning into beige adipocytes [27]. Additionally, thyroid hormones (T3/T4) potentiate thermogenesis through the dual mechanisms of central SNS activation and direct peripheral action on BAT. Cold exposure also increases the expression of 5-deiodinase type 2 (Dio2) and intracellular conversion from T4 to T3 to facilitate the induction of UCP1 and mitochondrial respiration [27–29]. TH also increases glucose uptake and lipolysis, providing fuel for β-oxidation.

#### Emerging pathways: UCP1-independent thermogenesis

Creatine cycling

Creatine kinase catalyzes the transfer of a phosphate group from ATP to creatine, generating phosphocreatine and ADP. The excess ADP formed drives thermogenic respiration in adipocytes [30]. This UCP1-independent mechanism operates through a futile creatine cycle. In 2015, Kazak et al*.*[31] proposed the role of creatine metabolism in thermogenesis. They demonstrated that the creatine cycle can sustain partial thermogenic capacity even in UCP1-knockout mice. Mitochondrial tissue-nonspecific alkaline phosphatase (TNAP) contributes to the futile creatine cycle by hydrolyzing phosphocreatine, thereby mediating thermogenesis [32]. Bunk et al. [33] restored thermogenic capacity and cold tolerance in mice by reintroducing mitochondrial CKB into brown adipocytes that were co-deficient for UCP1 and creatine kinase b (CKB). This rescue depended on the presence of TNAP. In contrast, simultaneously knocking out TNAP and UCP1 resulted in severe cold intolerance. This discovery challenges conventional perspectives, offering novel insights into the function and metabolism of BAT, with significance for conditions like obesity or aging (where UCP1 activity is generally diminished).

Calcium cycling

Calcium (Ca^2^⁺) cycling is a UCP1-independent thermogenic mechanism that primarily operates through futile energy expenditure via active Ca^2^⁺ transport and subsequent leakage in cells. Futile sarcoplasmic reticulum calcium ATPase (SERCA) pump activity induced by sarcolipin (SLN) binding can increase heat production and energy expenditure in muscles [34, 35]. Bal et al. [36] validated these findings using SLN-deficient mouse models, demonstrating that SLN-SERCA interaction promotes SERCA uncoupling, thereby increasing ATP hydrolysis and thermogenic capacity. Moreover, in the absence of UCP1, beige adipocytes promote glycolysis, tricarboxylic acid (TCA) cycle metabolism, and pyruvate dehydrogenase activity to dynamically consume glucose and generate ATP, thereby facilitating ATP-dependent thermogenesis through Ca^2^⁺ cycling [37]. This metabolic rewiring toward glucose oxidation highly depends on the SERCA2b-RyR2 pathway, and this increase significantly alters systemic metabolism by promoting the oxidation of carbohydrates. In another study on adipose tissue [38], the newly identified endoplasmic reticulum membrane peptide C4orf3/ALN was found to function as a “molecular resistor.” This peptide binds to SERCA2b and specifically decreases the energetic efficiency of calcium ion transport (rather than simply inhibiting it), thereby promoting the uncoupling of ATP hydrolysis and heat production. Knocking out C4orf3 impairs this thermogenic capacity in adipose tissue, leading to obesity and insulin resistance in mice. From SLN in muscle to C4orf3/ALN in fat, these tissue-specific regulators of SERCA form a key network for UCP1-independent thermogenesis, playing an important and synergistically compensatory role in cold adaptation and energy homeostasis in the body.

Lipid cycling

The esterification-lipolysis cycle of fatty acids is a crucial UCP1-independent thermogenic mechanism in adipocytes. This process involves repeated hydrolysis and re-esterification of TGs, and ATP is consumed to generate heat while activating endoplasmic reticulum (ER) stress-related pathways to further increase energy expenditure [39]. Oeckl et al. [40] found that the futile ATP-consuming cycle of TG lipolysis and FA re-esterification contributes to NST in brown adipocytes of UCP1 knockout (UCP1 KO) mice and promotes their survival in cold environments. Natarajan et al. [41] discovered that chronic treatment with β3-adrenergic agonist activates futile lipid cycling and increases energy expenditure in WAT of aged mice. Thus, stimulation of lipolysis and futile lipid cycling represents a potential therapeutic target for ameliorating age-related metabolic dysfunction.

Proton leak

Mitochondria can cycle protons (H^+^) independent of UCP1 [42]. The mitochondrial ADP/ATP carrier (AAC) is a major transport protein found in the inner mitochondrial membrane. It exchanges mitochondrial ATP for cytosolic ADP and controls ATP production in cells [43]. Additionally, the increase in the leakage of H^+^ ions is associated with cysteine oxidation in AAC. Through the modification of cysteine, maintaining the ADP/ATP transport function of AAC is crucial for developing anti-obesity therapeutic strategies that target AAC-dependent thermogenesis [44].

Peroxisome-driven branched-chain fatty acid oxidation cycle

In brown and beige adipose tissue, peroxisomes can drive thermogenesis through a “monomethyl branched-chain fatty acid (mmBCFA) synthesis – β-oxidation” cycle. Liu et al. [45] found that mmBCFAs are synthesized by fatty acid synthase (FASN) using short-branched acyl-CoA derived from the catabolism of branched-chain amino acids (BCAAs) and are subsequently oxidized in peroxisomes by acyl-CoA oxidase 2 (ACOX2), an ATP-consuming process that generates heat. Using knockout and overexpression models, the researchers demonstrated that loss of ACOX2 in adipose tissue impairs thermogenesis and exacerbates diet-induced obesity and insulin resistance, while the overexpression of ACOX2 promotes thermogenesis and improves metabolic homeostasis, even in the absence of UCP1. This was the first study to establish a key role for peroxisomes in the regulation of energy balance through the mmBCFA metabolic cycle, which operates independently of UCP1 and is considerably enhanced by cold exposure or cAMP signaling activation, providing a key compensatory mechanism in UCP1-deficient individuals.

## Progress in the study of natural thermogenic substances

### Traditional Chinese medicine and its active ingredients

#### *Panax ginseng* C. A. Mey.

Ginsenoside is the active ingredient in *P. ginseng* and can significantly increase heat production and improve cold tolerance. Rb_1_ is a key ginsenoside for cold tolerance and can even improve the reduction of heat production caused by age factors [46]. Xue et al. [47] demonstrated that the ginsenoside Rc attenuates cold-induced myocardial injury by suppressing the production of inflammatory cytokines and the apoptosis of cardiomyocytes via the activation of SIRT1, leading to a decrease in inflammatory responses. A study published in the 《BMJ》 demonstrated that ginseng extract promotes the development of thermogenic adipocytes by modulating gut microbiota metabolism. The extract increases the production of myristic acid by *Enterococcus faecalis*, which subsequently activates BAT and stimulates beige adipogenesis [48]. 

#### *Acanthopanax senticosus* (Rupr.etMaxim.) Harms

*Acanthopanax* extract enhances cold acclimatization by modulating physiological responses to cold exposure. *Acanthopanax* complex liquid upregulates superoxide dismutase (SOD) activity and cyclic adenosine monophosphate (cAMP) levels while suppressing the accumulation of malondialdehyde (MDA) in the membranes of erythrocytes. These biochemical changes improve energy metabolism and microcirculatory function, as indicated by high peripheral temperatures and enhanced vascular response index (VRCI), thereby promoting cold adaptation in animal models and human subjects [49].

#### *Rhodiola rosea* L.

*Rhodiola rosea* L. (RRL) is a traditional Chinese herb commonly referred to as “Tibetan ginseng”. It is widely used as a dietary supplement, medicinal agent, and functional food for high-altitude adaptation [50]. Rosavin, a characteristic bioactive constituent of RRL, has multiple pharmacological effects, including antioxidant, hypolipidemic, analgesic, anti-fatigue, and immunomodulatory activities. RRL modulates specific and nonspecific immune functions under hypothermic conditions, significantly enhancing the nonspecific resistance of the body to harmful stimuli while accelerating cold acclimatization in humans. Animal studies have found that RRL administration mitigates cold stress-induced metabolic alterations in rats, ameliorating metabolic disorders through the modulation of adrenal hormone secretion and subsequent regulation of the metabolism of the TCA cycle [51].

#### *Hypericum perforatum* L.

Hyperforin (HPF), a bioactive phytochemical derived from *Hypericum perforatum*, has significant metabolic regulatory effects in obese models. Biochemical analyses revealed that HPF binds to dihydrolipoamide S-acetyltransferase (Dlat) in adipose tissue, subsequently activating the AMPK/PGC-1α/Ucp1 signaling pathway. This activation upregulates the expression of thermogenic genes (including *UCP1*) in high-fat diet-induced obese mice and leptin-deficient mutant mice. Moreover, HPF treatment increases the abundance of proteins in the mitochondrial respiratory chain in subcutaneous white adipose tissue (sWAT) and BAT, while increasing the consumption of whole-body oxygen [52].

#### *Coptis chinensis* Franch.

Berberine (BBR), a quaternary ammonium alkaloid derived from *Rhizoma Coptidis*, is the primary antimicrobial constituent of this traditional Chinese medicinal plant. Zhang et al. [53] demonstrated that BBR upregulates UCP1 and the expression of other thermogenic genes in both WAT and BAT, as well as in primary adipocytes, through the activation of the AMPK/PGC-1α pathway, thereby enhancing thermogenesis in adipose tissue and cold tolerance. Moreover, Wu et al. [54] found that the administration of chronic BBR epigenetically stimulates the differentiation of brown adipocytes and thermogenic activity in BAT via the AMPK-PRDM16 axis, resulting in an increase in systemic energy expenditure and improvement in thermoregulation.

#### Compound Chinese medicines

Compound Chinese herbal formulations exhibit higher therapeutic efficacy compared to single-herb preparations due to synergistic interactions among multiple medicinal components. These formulations effectively prevent and treat cold-induced organ injuries while minimizing adverse effects. Lv et al. [55] developed a composite formulation that considerably increased catalase and SOD levels, enhanced total antioxidant capacity, and stabilized hepatic mitochondrial function and energy metabolism, together conferring cytoprotective effects. Similarly, Jin et al. [56] demonstrated that a medicinal food compound significantly increased core body temperature in acutely cold-exposed rats. This formulation normalized hormone levels in the HPO-axis and HPA-axis while upregulating thermogenic proteins (PGC-1α and UCP1) in BAT, thereby increasing thermogenesis in BAT and overall cold resistance (Table 1).

**Table 1:** Thermogenic effects of traditional Chinese medicine

Types	Active ingredients	Models	Impacts/effects	Refs
*Panax ginseng* C. A. Mey.	Ginsenoside, Rb_1_	SD mice	↑thermogenesis	[46]
	Ginsenoside, Rc	Rats	↓inflammation ↓myocardial injury ↑SIRT1	[47]
	Ginseng extract	Db/db mice	↓body weight ↑beige formation ↑BAT activity ↑energy expenditure	[48]
*Acanthopanax senticosus (Rupr.etMaxim.) Harms*	Extractum Acanthopanacis Senticosi	Wistar mice, Men	↑energy metabolism ↑blood flow ↑thermogenesis	[49]
*Rhodiola rosea* L.	/	Wistar mice	↓metabolic disorder	[51]
*Hypericum perforatum* L.	Hyperforin	C57BL/6 J, Ob/ob and DIO mice	↓ body weight and fat accumulation ↑UCP1、thermogenesis ↑glucose tolerance ↑iWAT browning and BAT activity	[52]
*Coptis chinensis* Franch.	Berberine	Db/db mice,C3H10T1/2 cells	↓body weight ↑glucose tolerance ↑thermogenesis, energy expenditure ↑UCP1, PGC‑1α ↑WAT and BAT activity ↑brown adipogenesis	[53, 54]
		Mildly overweight NAFLD patients, C57BL/6 J and DIO mice		
Compound Chinese medicine	/	SD mice	↑energy metabolism ↑antioxidant ability	[55]
			↑thermogenesis ↑PGC‑1α, UCP1	[56]

### Nutrients

Diet-induced thermogenesis (DIT), also known as the thermic effect of food (TEF), is defined as increased energy expenditure in the human body after food intake. This process involves several physiological activities, such as the digestion, absorption, transport, metabolism, and storage of food, and is a significant component of total energy expenditure (TEE), accounting for approximately 10% of daily energy expenditure [57]. This diet-initiated thermogenic mechanism is physiologically closely associated with another important form of thermogenesis in humans—cold-induced thermogenesis. For example, BAT not only mediates NST via uncoupling protein 1 (UCP1) to maintain body temperature in cold environments but also participates in the regulation of postprandial energy metabolism [58]. The degree of its activation directly influences overall energy expenditure levels. Therefore, optimizing dietary structure may help synergistically activate and promote multiple thermogenic pathways, including diet-induced and cold-induced thermogenesis, thereby improving cold tolerance while promoting overall metabolic health.

#### Proteins, amino acids, or amino acid-like substances

High-protein diets regulate energy metabolism and thermogenic processes through multiple pathways, particularly by activating brown and beige/BRITE adipose tissues [59]. Murine studies revealed that high dietary protein content significantly increased body surface temperature and upregulated thermogenic markers (*UCP1*,* PGC1-α*, and *DIO2*) in BAT [60]. Human clinical trials conducted by Maliszewska et al. [61] demonstrated that protein source differentially influences WAT browning and increases the thermogenic capacity of BAT.

Amino acids are the fundamental constituents of proteins and exhibit diverse thermogenic properties. Tyrosine (Tyr) serves as a precursor for catecholamine neurotransmitter synthesis and facilitates the production and release of catecholamines in the central and peripheral nervous systems; its metabolism is particularly sensitive to cold stress. Meng et al. [62] found that tyrosine-cysteine co-administration significantly decreased serum total cholesterol and low-density lipoprotein (LDL) cholesterol levels in cold-exposed mice. This treatment concurrently upregulated the expression of UCP1, PPAR-γ, and PPAR-α. These metabolic improvements increased the hepatic thermogenic capacity and conferred hepatoprotective effects in the murine model.

Tryptophan (Trp), the metabolic precursor of serotonin (5-hydroxytryptamine, 5-HT), functions as a key neurotransmitter in the central nervous system. Its cerebral concentration modulates neural activity, subsequently influencing the secretion of hypothalamic prohormones. This neuroendocrine cascade stimulates pituitary-mediated release of triiodothyronine (T3), thyroxine (T4), and glucocorticoids, ultimately promoting thermogenesis and increasing the metabolic rate. Yang et al. [63] demonstrated that the combined administration of choline, tyrosine, and Trp increased caloric production by 5.43–173.46% in littermates, which indicated that these compounds can acutely increase the basal metabolic rate and the thermogenic capacity.

Taurine, an endogenous cytoprotective substance, exerts antioxidant effects by scavenging free radicals, thereby mitigating cold stress-induced impairments in cerebral and systemic functions in a dose-dependent manner. Kuwahara et al. [64] demonstrated in animal studies that taurine supplementation preferentially increases the activity of sympathetic nerves, thereby protecting cardiovascular and autonomic functions in cold-exposed rats. Moreover, dietary taurine supplementation improves muscular performance, cardiac function, hepatic activity, and energy metabolism in adipose tissue [65].

Carnitine, a quaternary ammonium compound biosynthesized from lysine and methionine, plays a key role in fatty acid metabolism and energy production. l-carnitine preferentially accumulates in BAT, promoting both lipogenesis and thermogenesis in caprine brown adipocytes. A recent study revealed that maternal l-carnitine supplementation promotes the development of BAT and thermogenic capacity in newborn goats, providing novel mechanistic insights into their thermoregulatory adaptation to cold exposure [66].

#### Fats

High-fat dietary intake facilitates cold acclimatization and increases cold resistance [67, 68]. Short-term consumption of foods containing approximately 30% fat under cold exposure improves cold tolerance in combat personnel without inducing lipid metabolism disorders, whereas higher fat content (up to 45%) may have adverse effects. The combined supplementation of high-fat meals (30% fat thermogenic ratio) with antioxidants (VC, VE, and Zn) accelerates cold adaptation and improves cold tolerance [69]. Kus et al. [70] demonstrated enhanced systemic lipid oxidation in cold-exposed obesity-resistant A/J mice compared to obesity-prone C57BL/6 J mice, and identified that a high-fat diet induces oxidative muscle thermogenesis through the leptin-AMPK signaling axis.

Omega-3 polyunsaturated fatty acids (PUFAs) have significant effects on BAT activation and thermogenic regulation. Fish oil supplementation promotes WAT browning, upregulating UCP1 expression and enhancing BAT thermogenic capacity. Kim et al. [71] further elucidated that eicosapentaenoic acid (EPA) and docosahexaenoic acid (DHA) in fish oil stimulate the SNS through the activation of transient receptor potential vanilloid 1 (TRPV1) in gastrointestinal afferent nerves, resulting in the upregulation of UCP1 in both interscapular BAT and inguinal WAT. Bile acids (BAs) are metabolic regulators that play important roles in lipid digestion, energy homeostasis, and glucose and lipid metabolism. Watanabe et al*.*[72] found that administering BA in mice activates TGR5 in brown adipocytes, subsequently inducing type 2 deiodinase activity and promoting thermogenesis, which collectively ameliorates obesity and insulin resistance.

Short-chain fatty acids (SCFAs), including acetate, propionate, and butyrate, enhance BAT thermogenesis and WAT browning. Butyrate acts as a thermogenic molecule by upregulating LSD1, UCP1, and PGC-1α, thereby stimulating thermogenesis in BAT and scWAT [73, 74]. Hu et al*.* [75] reported that acetate treatment increases the expression of PGC-1α and UCP1 in brown adipocytes, along with alterations in mitochondrial morphology. Propionate contributes to adaptive thermogenesis by activating the SNS via GPR41, a Gi/o protein-coupled SCFA receptor, which enhances energy expenditure, thereby providing indirect evidence for its role in thermoregulation [76].

#### Carbohydrates

Carbohydrates affect thermogenesis through multiple mechanisms. Comparative studies have demonstrated that individuals consuming high-carbohydrate meals exhibit approximately 10% greater oxygen uptake and over 100% higher blood flow compared to those consuming high-fat meals, with preferential perfusion of BAT and enhanced thermogenic activity [77]. Both brown and beige adipocytes activate UCP1-mediated thermogenesis in response to cold exposure by catabolizing stored lipids and carbohydrates [78].

#### Vitamins

During the early phase of acclimatization to cold, the increase in lipid peroxidation can lead to the accumulation of its byproducts. Vitamin E (VE) is a potent biological antioxidant and can effectively inhibit the peroxidation of unsaturated fatty acids, eliminate lipid peroxidation products, protect cellular membranes from oxidative damage, and increase energy metabolism to mitigate cold stress [79]. Supporting this, Wang et al. [80] showed that VE supplementation increases Na^+^/K^+^-ATPase activity in human erythrocyte membranes while reducing plasma MDA levels, thereby suppressing lipid peroxidation and improving cold tolerance.

Vitamin C (VC) increases immune function by promoting the production of antibodies, increasing the phagocytic activity of leukocytes, and improving disease resistance and cold tolerance. VC also has protective effects on vascular function. Jin et al. [81] demonstrated that VC supplementation in cold-stressed mice significantly improved histological architecture and serum biochemical parameters in renal and testicular tissues, indicating that it has substantial protective effects against cold-induced damage.

Vitamin B2 supplementation potentiates the expression of T4-induced flavoprotein enzyme, improves cold adaptation capacity, and decreases circulating cortisol levels in rats, thereby attenuating their sensitivity to acute cold stress [82]. Moreover, vitamin B2 acts synergistically with VE and iron to enhance energy metabolism and participate in hematopoietic processes, collectively promoting thermogenesis for cold protection.

Vitamin D plays crucial roles in multiple physiological processes, including energy metabolism, antioxidant defense, adipocyte differentiation, and apoptosis [83]. Therefore, vitamin D deficiency disrupts the secretion of adipocytokines, metabolic homeostasis, lipid storage, lipogenesis, thermogenic regulation, inflammatory responses, and oxidative stress balance. Nimitphong et al. [84] demonstrated that vitamin D increases thermogenesis through the activation of BAT. Additionally, vitamin D insufficiency is associated with the downregulation of UCP1 expression and the suppression of AMP-activated protein kinase/sirtuin 1 (AMPK/SIRT1) signaling in WAT [85].

Retinoic acid (RA), an active metabolite of vitamin A, functions as a potent activator of thermogenesis in BAT. RA upregulates the expression of UCP1, promotes lipolysis, improves mitochondrial function, and induces brown-like morphological changes in WAT adipocytes. Deis et al. [86] further revealed that lipocalin-2 (Lcn2), a lipid transport protein, modulates RA signaling by facilitating its binding to RA receptor-alpha (RAR-α) and subsequent nuclear translocation, ultimately upregulating the expression of UCP1 to promote both WAT browning and BAT thermogenesis.

#### Mineral substance

Cold stress triggers a cascade of neuroendocrine adaptations characterized by an increase in metabolic activity and greater demand for essential trace elements (Na, Fe, Cu, Zn, and I) to facilitate cold adaptation. Zinc supplementation in cold-exposed rats significantly attenuates hepatic and skeletal muscle zinc depletion induced by acute cold exposure, extending the duration of survival by 15.9% [87]. Similarly, high iron availability enhances the activity of iron-dependent enzymes, thereby improving tolerance to cold stress and allowing rats to maintain their core body temperature under hypothermic conditions [88].

#### Composite nutritional preparation

Complex nutrient formulations increase stress adaptation and cold resistance through synergistic interactions of multiple micronutrients and natural bioactive compounds. Liu et al. [89] demonstrated that supplementation with an optimized combination of VC, VE, and glutamine (Gln) significantly reduces serum NE levels while enhancing glutathione peroxidase (GSH-PX) and SOD activities. This formulation effectively downregulates the expression of heat shock protein 70 (HSP70), improves cellular repair capacity under cold stress, and increases total antioxidant capacity in rats. These nutrient complexes preserve the ultrastructural integrity of skeletal muscle cells in cold-exposed rats by maintaining mitochondrial and myofibrillar morphology, thereby sustaining intracellular ATP levels [90]. Jin et al. [91] developed a compound nutrient preparation that upregulates the expression of follicle-stimulating hormone receptor (FSHR) and luteinizing hormone receptor (LHR) proteins in the ovaries and uteri of cold-stressed female rats. This preparation increases energy metabolism and related enzymatic activities while maintaining hormonal homeostasis and the function of the hypothalamic-pituitary-ovarian (HPO) axis under cold exposure (Table 2).

**Table 2:** Nutrient-induced thermogenesis

Types	Active ingredient	Models	Impacts/effects	Refs
Proteins	/	C57BL/6 J mice	↑thermogenesis ↑WAT and BAT stores ↑PGC‑1α、UCP1	[60]
		Healthy men	↑WAT browning and BAT activity	[59, 61]
Amino acids	Tyrosine and cysteine	C57BL/6 J mice	↓ body weight ↑thermogenesis ↑PGC‑1α, UCP1	[62]
	Tryptophan and tyrosine	Wistar mice	↑thermogenesis	[63]
	Taurine	Rats	↑SNS	[64]
Oramino acid-like substance	l-carnitine	Chuangzhong black goats	↑thermogenesis ↑BAT activity	[66]
Fats	High fat	SD mice	↑thermogenesis ↑lipid metabolism	[68, 69]
		C57BL/6 J mice and A/J mice	↑thermogenesis ↑UCP1	[70]
	Omega-3 fatty acids	Mice	↑WAT browning and BAT activity ↑UCP1	[71, 92]
	Bile acids	Mice	↑energy expenditure ↑thermogenesis	[72]
	Butyrate	Mice	↑thermogenesis ↑PGC‑1α、UCP1	[73]
		C57BL/6 J mice	↑energy expenditure ↑PGC‑1α ↑insulin sensitivity ↑mitochondrial function	[74]
	Acetate	C57BL/6 J mice, brown adipocyte cells	↑PGC‑1α, UCP1 ↑mitochondrial biogenesis	[75]
	Propionate	Mice	↑SNS ↑energy expenditure	[76]
Carbohydrates	/	Mice	↑thermogenesis and blood flow	[77]
Vitamin	Vitamin E	Healthy men	↓lipid peroxidation	[80]
	Vitamin C	Kunming mice	↑hepatorenal function	[81]
	Vitamin B2	Wistar mice	↓stress sensitivity ↑body weight	[82]
	Vitamin D	Obese rats	↓body weight ↑WAT browning and BAT activity	[84, 85]
	Retinoic acid	Lcn2-deficient mice	↑WAT browning and BAT activity ↑UCP1, thermogenesis	[86]
Mineral substance	Zn	SD mice	↑survival rate	[87]
	Fe	SD mice	↑cold tolerance	[88]
Composite nutritional preparation	/	SD mice	↑antioxidant ability ↑energy expenditure	[89–91]

### Food active ingredients

Phytochemicals include different types of structurally distinct plant-derived bioactive compounds with health-promoting properties, including flavonoids, organosulfides, polyphenols, saponins, dietary fibers, and specific plant polysaccharides [93, 94]. These bioactive compounds exert multiple beneficial effects in cold resistance through thermogenic properties, thermoregulatory modulation, enhancement of metabolic activity, and provision of essential micronutrients.

#### Polymethoxylated flavones (PMFs)

Polymethoxylated flavones (PMFs) are a distinct subclass of flavonoids predominantly found in *Citrus* species and exert significant thermogenic effects. In *Citrus aurantium* L.-derived PMF-treated mice, the mitochondrial biogenesis regulators TFAM and NRF1/2 are strongly activated, resulting in an increase in mitochondrial biosynthesis, greater BAT activity, and induction of browning of Iwat [95]. Additionally, cold tolerance assays revealed that PMF supplementation effectively maintained core body temperature and augmented NST capacity.

#### Tea polyphenols

Tea polyphenols (TPs), the predominant bioactive constituents in tea, consist of phenolic compounds and their derivatives, primarily classified into catechins and flavonoids. Dulloo et al. [96] demonstrated the thermogenic potential of green tea extract in humans, showing that consumption of catechin-enriched and caffeine-enriched extract increased 24 h energy expenditure (EE) by 4% while enhancing fat oxidation. Ji et al. [97] demonstrated that dietary tea polyphenol supplementation significantly attenuates cold-induced increase in Evans blue extravasation and cerebral water content, suggesting that it has a neuroprotective role against cold exposure-induced dysfunction of the central nervous system (CNS). Zhou et al. [98] revealed that the supplementation of epigallocatechin gallate (EGCG) upregulates the expression of UCP1 in brown adipocytes, thereby increasing thermogenic capacity. Moreover, their cold tolerance tests at 4 °C demonstrated the ability of EGCG to enhance cold adaptation through greater heat production. An acute and chronic human intervention trial revealed that oral intake of catechins (containing caffeine) significantly increases whole-body energy expenditure [99]. This effect is closely associated with the activity of BAT, and long-term intake can further increase cold-induced thermogenesis, suggesting that catechins may promote thermogenesis by activating and recruiting BAT.

#### Capsaicin and capsaicinoids

Transient receptor potential vanilloid 1 (TRPV1)-expressing progenitor cells in BAT undergo thermogenic differentiation in response to cold exposure [100, 101]. Capsaicin serves as a potent TRPV1 agonist. When capsaicin is administered orally, it activates gastrointestinal TRPV1-expressing sensory neurons. This stimulation enhances sympathetic outflow to BAT, resulting in the rapid thermogenic activation of BAT and subsequent increase in systemic energy expenditure (EE) [102].

#### Caffeine

Velickovic et al. [103]*.* systematically examined the effects of caffeine on thermogenesis in BAT through in vitro and in vivo approaches. They found that caffeine upregulates key thermogenic markers, including PGC-1α and UCP1, while promoting mitochondrial biogenesis in brown adipocytes. The administration of caffeine significantly increased UCP1 and the expression of thermogenic genes in BAT, concurrently inducing sWAT browning and inhibiting iBAT whitening, indicating that caffeine has potent BAT activation potential [104].

#### Quercetin

Quercetin is a bioactive flavonol that upregulates thermogenic gene expression in adipose tissues of high-fat diet-fed mice, including *UCP1, PGC-1α,* and mitochondrial transcription factor A (*Tfam*). It also enhances the expression of β3-adrenergic receptors. Quercetin promotes AMP-activated protein kinase (AMPK)-dependent SIRT1 activation, leading to the deacetylation of PGC-1α and subsequent upregulation of the expression of PGC-1α and UCP1 [105].

#### Resveratrol

Resveratrol is a non-flavonoid polyphenolic compound abundant in grapes, berries, and red wine; it exhibits potent thermogenic effects. Resveratrol upregulates the expression of the mRNAs and proteins of key brown adipocyte markers, including UCP1, Prdm16, and PGC-1α [106]. Mice fed a high-fat (HF) diet supplemented with 0.1% resveratrol showed high expression of UCP1 and brown-like morphological changes in iWAT. Resveratrol functions as a prebiotic-like compound and modulates gut microbiota composition, thereby enhancing thermogenesis in adipose tissue [107]. An intervention study revealed that 0.4% resveratrol supplementation for four weeks significantly reduced adiposity, promoted WAT browning, and ameliorated gut microbiota dysbiosis in mice fed an HF diet. Moreover, combined administration of resveratrol and quercetin synergistically induced browning in perirenal WAT and upregulated the expression of UCP1 in iBAT [108].

#### Anthocyanins and their metabolites

Cyanidin-3-glucoside (C3G), a predominant anthocyanin constituent in mulberry, has several metabolic benefits in obese mice. The administration of C3G increases energy expenditure, attenuates weight gain, maintains glucose homeostasis, ameliorates hepatic steatosis, and improves tolerance to cold. C3G increases BAT thermogenic capacity and induces WAT browning through mitochondrial biogenesis and the expression of UCP1 in both BAT and sWAT [109]. Vanillic acid (VA), a major microbial metabolite of anthocyanins, has similar thermogenic properties. VA supplementation decreases adiposity, stimulates WAT browning, and activates thermogenesis in BAT, particularly in the inguinal WAT of mice fed an HF diet [110].

#### Menthol

Menthol, a cyclic monoterpene alcohol (also known as menthol camphor), is either synthetically produced or naturally derived from *Mentha* species. Jiang et al. [111]*.* demonstrated that menthol upregulates the expression of thermogenic genes (*UCP1 *and *PGC-1α*) in white adipocytes through transient receptor potential melastatin 8 (TRPM8)-mediated activation of the protein kinase A (PKA) signaling pathway. Menthol administration attenuated high-fat diet (HFD)-induced weight gain and insulin resistance in obese mice. Topical application of menthol activated TRPM8 channels, triggering NST and behavioral thermogenic responses, ultimately increasing the core body temperature [112]. In 2025, Wakabayashi et al. [113] revealed the relationship between human BAT activity and menthol-induced thermogenesis (via activation of the transient receptor potential melastatin-8 channel). They observed that menthol significantly increased energy expenditure and fat oxidation in individuals with high BAT activity, confirming that human thermogenic responses and BAT activity have a direct relationship.

#### Curcumin

Curcumin, a natural polyphenolic flavonoid derived from turmeric (*Curcuma longa*), has significant thermogenic properties. Lone et al. [114] demonstrated that curcumin induces the differentiation of beige adipocytes in both 3T3-L1 and primary mouse white adipocytes, upregulating key thermogenic markers including UCP1, PGC-1α, and PRDM16. This process is mediated through the activation of AMPK, concomitant with a decrease in lipid accumulation. Complementary research by Li et al. [115] revealed that ginger-derived curcumin increases systemic thermogenesis by increasing circulating thyroid hormone levels and ameliorating cold-induced thyroid tissue damage in mice.

#### Ellagitannins

Urolithin A (UA), a bioactive metabolite derived from ellagitannins (ETs) through gut microbial transformation, is abundant in pomegranates and certain other fruits and nuts. Pomegranates are a rich source of ETs that undergo intestinal metabolism to yield UA. Xia et al. [116] demonstrated that the administration of UA significantly increased energy expenditure in mice by activating thermogenesis in BAT and promoting WAT browning. This thermogenic effect was attributed to high T3 levels in BAT and inguinal adipose depots (Table 3).

**Table 3:** Thermogenic effects of food active ingredients

Types	Active ingredient	Models	Impacts/effects	Refs
Polymethoxylated flavones	/	C57BL/6J mice	↑thermogenesis ↑iWAT browning and BAT activity ↑mitochondrial biogenesis	[95]
Tea polyphenols	Catechin polyphenols and caffeine	Healthy men	↑thermogenesis ↑energy expenditure ↑fat oxidation	[96]
		SD mice	↓blood–brain barrier	[97]
		Healthy men	↑BAT activity ↑ thermogenesis ↑energy expenditure	[99]
	Epigallocatechin gallate	C57BL/6J mice	↓neuroinflammation ↓lipid accumulation ↑BAT thermogenesis ↑energy expenditure ↑PGC‑1α、 UCP1	[98]
Capsaicin and capsinoids	/	Healthy men	↑BAT activity ↑energy expenditure	[100]
Catechins	/	Healthy men and women	↑thermogenesis ↑UCP1 ↑mitochondrial biogenesis	[103]
		Obese mice	↓body weight ↑glucose tolerance ↑sWAT browning ↑mitochondrial biogenesis	[104]
Quercetin	/	C57BL/6 mice	↑mitochondrial biogenesis ↑PGC‑1α、 UCP1	[105]
Resveratrol	/	Female mice	↓body weight ↑iWAT browning ↑energy expenditure ↑UCP1, PRDM16	[106]
		C57BL/6J mice	↓alteration of intestinal flora ↓lipid accumulation ↑WAT browning	[107]
		Rats	↓body weight ↑UCP1 ↑pWAT browning	[108]
Anthocyanins	Cyanidin-3-glucoside	Db/db mice	↓body weight ↑energy expenditure ↑sWAT browning and BAT activity ↑UCP1 ↑mitochondrial function	[109]
Anthocyanin metabolites	Vanillic acid	C57BL/6J mice	↓body weight ↑thermogenesis ↑glucose tolerance ↑iWAT browning and BAT activity	[110]
Menthol	/	C57BL/6J mice, white adipocytes	↑thermogenic gene expression ↑Ca^2+^ ↑WAT browning	[111]
		C57BL/6J mice	↑thermogenic ↑SNS	[112]
		Healthy men	↑energy expenditure ↑fat oxidation ↑thermogenesis	[113]
Curcumin	/	3T3-L1 adipocytes ,primary white adipocytes	↑UCP1, PGC‑1α, PRDM16 ↑mitochondrial biogenesis	[114]
		Kunming mice	↑thermogenic ↑thyroid hormones	[115]
Ellagitannins	Urolithin A	C57BL/6 mice, Primary adipocytes	↑WAT browning and BAT activity ↑energy expenditure ↑thyroid hormones ↑glucose tolerance	[116]

### Other small molecules

Succinic acid (succinate), a key TCA cycle intermediate, functions as a potent activator of thermogenesis. Researchers at Dana-Farber Cancer Institute demonstrated that intravenous administration of carbon isotope-labeled succinate preferentially accumulates in BAT, where it promotes lipolysis and activates thermogenesis through its metabolic action as a TCA cycle intermediate [117].

Mirabegron, a selective β3-adrenergic receptor (β3-AR) agonist clinically approved for treating overactive bladder syndrome, has significant metabolic effects. It activates thermogenesis in BAT and stimulates lipolysis in WAT. Both in vivo and in vitro studies have confirmed that mirabegron upregulates the expression of UCP1, induces inguinal WAT (iWAT) browning, improves glucose homeostasis, and prevents HFD-induced obesity [118]. However, its clinical application is limited because it increases blood pressure in a dose-dependent manner.

Ephedrine, a naturally occurring sympathomimetic amine derived from *Ephedra* species, elicits thermogenic effects through multiple pharmacological mechanisms. It is a non-selective adrenergic receptor agonist that binds to both α-AR and β-AR, increasing SNS activity to stimulate thermogenesis, lipolysis, and glucose metabolism. Carey et al. [119] observed that acute administration of ephedrine activates BAT thermogenic activity in healthy lean adults. However, its clinical application needs to be monitored carefully due to potential cardiovascular (elevated blood pressure and tachycardia) and neurological (insomnia, headache) adverse effects, as well as gastrointestinal disturbances, including nausea.

Inosine is an important regulator of energy metabolism in both brown and beige adipocytes. Willemsen et al. [120] demonstrated that inosine activates the cAMP/PKA signaling pathway, thereby increasing thermogenic capacity in brown adipocytes. Moreover, the administration of inosine in mice significantly increased BAT-dependent energy expenditure and promoted WAT browning. The protein equilibrative nucleoside transporter 1 (ENT1) serves as a key modulator of intracellular inosine levels in adipocytes.

Irisin is a myokine secreted by skeletal muscle, identified in 2012. It functions as a peroxisome proliferator-activated receptor gamma coactivator 1α (PGC-1α)-dependent hormone. PGC-1α regulates mitochondrial biogenesis and oxidative metabolism across various types of cells, while also mediating the expression of UCP1 and BAT thermogenesis [20, 121]. Irisin can also induce browning of subcutaneous white adipocytes and UCP1-mediated thermogenesis [122]. Zhang et al. [123] demonstrated that irisin increases mitochondrial biogenesis in white adipocytes through the activation of the p38 MAPK and ERK pathways, resulting in the upregulated expression of UCP1 and PGC-1α.

Leptin, a peptide hormone encoded by the Lepob^ob^ gene, is predominantly secreted by WAT. It binds to hypothalamic leptin receptors (LepR), resulting in the suppression of appetite and an increase in energy expenditure, which collectively contribute to the loss of body weight [124, 125]. Under cold exposure, leptin stimulates thermogenesis through SNS activation, promoting BAT activation and WAT browning [126–128]. Dodd et al. [129] demonstrated that the synergistic action of leptin and insulin on hypothalamic neurons increases WAT browning and energy expenditure while reducing body weight in DKO mice (Table 4).

**Table 4:** Thermogenic effects of small molecules

Types	Models	Impacts/effects	Refs
Succinate	C57BL/6 mice, primary brown adipocyte	↑BAT thermogenic ↑UCP1	[117]
Mirabegron	C57BL/6 mice, 3T3-L1 adipocytes	↓ body weight ↑WAT browning ↑glucose tolerance ↑UCP1	[118]
Ephedrine	Young men	↑BAT activity	[119]
Inosine	Fat cells	↑thermogenic	[130]
	C57BL/6 mice, Primary human and murine adipocyte	↑energy expenditure ↑iWAT browning	[120]
Irisin	Healthy adults, primary adipocytes	↑White adipocyte browning ↑UCP1	[122]
	Primary adipocyte culture, 3T3-L1 preadipocytes, C57BL/6 mice	↑PGC‑1α, UCP1 ↑mitochondrial biogenesis	[123]
Leptin	C57BL/6J mice, Ob/ob mice	↑thermogenesis ↑UCP1	[126–128]
	Mice	↓body weight ↑WAT browning and BAT thermogenesis ↑energy expenditure	[129]

## Summary and outlook

Although thermogenic substances have significant potential in combating cold stress, research on these substances has several critical limitations: the complex influence of variations among individuals in cold adaptation (e.g., genetics, gut microbiota) on the dose–response relationships of these components, the scarcity of cross-seasonal and cross-regional studies, and the unclear dose–effect relationships of natural ingredients, all of which make it difficult to develop strategies for future application. This review focused on the thermogenic mechanisms of nutrients, traditional Chinese medicines, and dietary bioactive compounds. We integrated evidence from molecular, cellular, and whole-organism levels to elucidate the mechanism by which natural thermogenic substances maintain health under cold exposure. This review also provides theoretical support for developing specialized foods for cold regions, formulating anti-cold TCM prescriptions, and designing personalized nutritional interventions.

## Data Availability

No datasets were generated or analyzed during the current study.
